# Targeted deletion of *Ruvbl1* results in severe defects of epidermal development and perinatal mortality

**DOI:** 10.1186/s40348-021-00111-1

**Published:** 2021-02-12

**Authors:** Claudia Dafinger, Thomas Benzing, Jörg Dötsch, Bernhard Schermer, Max C. Liebau

**Affiliations:** 1 Department of Pediatrics, Faculty of Medicine and University Hospital Cologne, University of Cologne, Kerpener Str. 62, 50937 Cologne, Germany; 2 Department II of Internal Medicine, Faculty of Medicine and University Hospital Cologne, University of Cologne, Cologne, Germany; 3 CECAD, Faculty of Medicine and University Hospital Cologne, University of Cologne, Cologne, Germany; 4 Systems Biology of Ageing Cologne, University of Cologne, Cologne, Germany; 5 Center for Molecular Medicine, University of Cologne, Cologne, Germany

## Abstract

Epidermal development is a complex process of regulated cellular proliferation, differentiation, and tightly controlled cell death involving multiple cellular signaling networks. Here, we report a first description linking the AAA+ (ATPases associated with various cellular activities) superfamily protein Ruvbl1 to mammalian epidermal development. Keratinocyte-specific *Ruvbl1* knockout mice (*Ruvbl1*^fl/fl^*K14:Cre*^tg^) show a severe phenotype including dramatic structural epidermal defects resulting in the loss of the functional skin barrier and perinatal death. Thus, *Ruvbl1* is a newly identified essential player for the development of differentiated epidermis in mice.

**Supplementary Information:**

The online version contains supplementary material available at 10.1186/s40348-021-00111-1.

## Introduction

The development of the mammalian epidermis is a highly complex multistage process that involves a sequence of steps from epidermal specification to expansion and development of derivative appendages, e.g., hair follicles [1, 2]. For the precise regulation of epidermal development, several signaling pathways are involved closely regulating the spatiotemporal coordination of cellular proliferation, differentiation, and tightly controlled cell death [1, 3, 4]. Epidermal development has become a model to study developmental mesenchymal-epithelial interactions between epidermis and dermis, the specific mechanisms of embryonic wound healing without scarring and cellular mechanisms in rapidly proliferating and self-renewing tissues [1–4].

The two AAA+ (ATPases associated with various cellular activities) superfamily proteins Ruvbl1 (Pontin, Tip48) and Ruvbl2 (Reptin, Tip49) have their central roles in promoting cell proliferation and cell survival. They contribute to various cellular processes including chromatin remodeling (Tip60, Ino80, and Scrap) as well as DNA damage signaling and repair [5–13]. Ruvbl1 is, e.g., required for the assembly and function of multiple protein complexes including the Tip60 complex regulating the tumor suppressor protein p53 [8, 13, 14]. In addition, the Ruvbl proteins or protein complexes involving the Ruvbl proteins modulate transcriptional activity of, e.g., pro-proliferative factors like MYC and ß-catenin and play important roles in multiple steps of cell cycle progression and cellular metabolism [6, 8, 13]. We recently identified Ruvbl1 as a component of ciliary protein complexes in renal tubular, bronchial, and ependymal epithelial cells [15]. Ruvbl1 also seems to be essential for early embryogenesis in mice [15, 16], and it has been suggested that loss of murine stem cell populations may be due to apoptosis [16]. To study the significance of Ruvbl1 in rapidly proliferating and differentiating mammalian tissue in vivo, we generated a targeted knockout of *Ruvbl1* in murine epidermal cells. Here, we show that the embryonic loss of *Ruvbl1* in mouse epidermis leads to severe developmental defects, resulting in loss of skin architecture and skin barrier defects associated with perinatal death.

## Material and methods

### Mice

The generation of the *Ruvbl1*^fl^ mice was recently described [15]. K14:Cre (MGI: 2177413) expressing the Cre recombinase under the Keratin14 promotor in the epidermis has previously been described [17]. To generate epidermal-specific *Ruvbl1*-deficient mice, *Ruvbl1*^fl^ animals and K14:Cre animals were crossed on a C57Bl/6N background. The mice were housed according to standardized specific pathogen-free conditions in the animal facility of the University of Cologne. All matings and experiments were conducted in accordance with European, national and institutional guidelines, as approved by the State Office of North Rhine-Westphalia, Department of Nature, Environment and Consumer Protection (8.87-50.10.31.08.049 and 84-02.04.2013.A152).

For the preparation of the embryonic mice, the pregnant females were sacrificed by cervical dislocation. Tissue was processed by fixation in 4% formaldehyde and embedding in paraffin.

### Skin barrier assay

For examination of the skin barrier, intact E18.5 embryos were first dehydrated in increasing amounts of methanol (25%, 50%, 75%, 100%). After rehydration in decreasing methanol solutions (75%, 50%, 25%, 0%), the embryos were stained in 0.0125% toluidine blue for 1 min and finally washed in PBS.

### Histology

For histological analysis, embryonic tissue was cut in 3-μm-thick sections and stained with hematoxylin and eosin using standard techniques. Paraffin was removed from the embedded tissue by xylene treatment and rehydration in graded ethanol. Sections were then stained, firstly, with Mayer’s hemalum (Sigma-Aldrich) to visualize nuclei in blue, and secondly, with eosin (Carl Roth) to visualize cytoplasmic structures in red.

### Immunostainings

Embryonic tissue was sliced in 2-μm-thick sections and deparaffinated as described above. Antigen retrieval was achieved using heat-induced epitope retrieval and citrate buffer. For immunohistochemical staining, endogenous peroxidases and unspecific antibody binding sites were blocked by treatment with 3% H_2_O_2_ and the ABC Kit according to the manufacturer’s manual (VectorLabs). Primary antibodies were incubated overnight at 4°C in 1% BSA as indicated. After incubation with the biotin-coupled secondary antibody, antibody signals were visualized by developing the tissue in DAB solution (VectorLabs). Nuclear counterstaining was performed using Mayer’s hemalum reagent. For fluorescent staining, tissue was blocked in 5% normal donkey serum (Jackson ImmunoResearch) and 1% bovine serum albumin for 1 h at RT. Primary antibodies were incubated overnight at 4 °C as indicated. Tissue sections were incubated with fluorophore-coupled secondary antibodies and mounted in Prolong Diamond antifade containing DAPI (Invitrogen).

### Antibodies

The following antibodies were used: Ki67 (Abcam, 1:500), K14 (Abcam, 1:500), Ruvbl1 (ProteinTech, 1:500), p53 (Leica Biosystems, 1:500), CC3 (Cell Signalling, 1:200), yH2Ax (Cell Signalling, 1:500), ß-cat (Cell Signalling, 1:500), cMyc (Cell Signalling, 1:500).

## Results

### Epidermal *Ruvbl1* knockout results in perinatal mortality

**Fig. 1:**
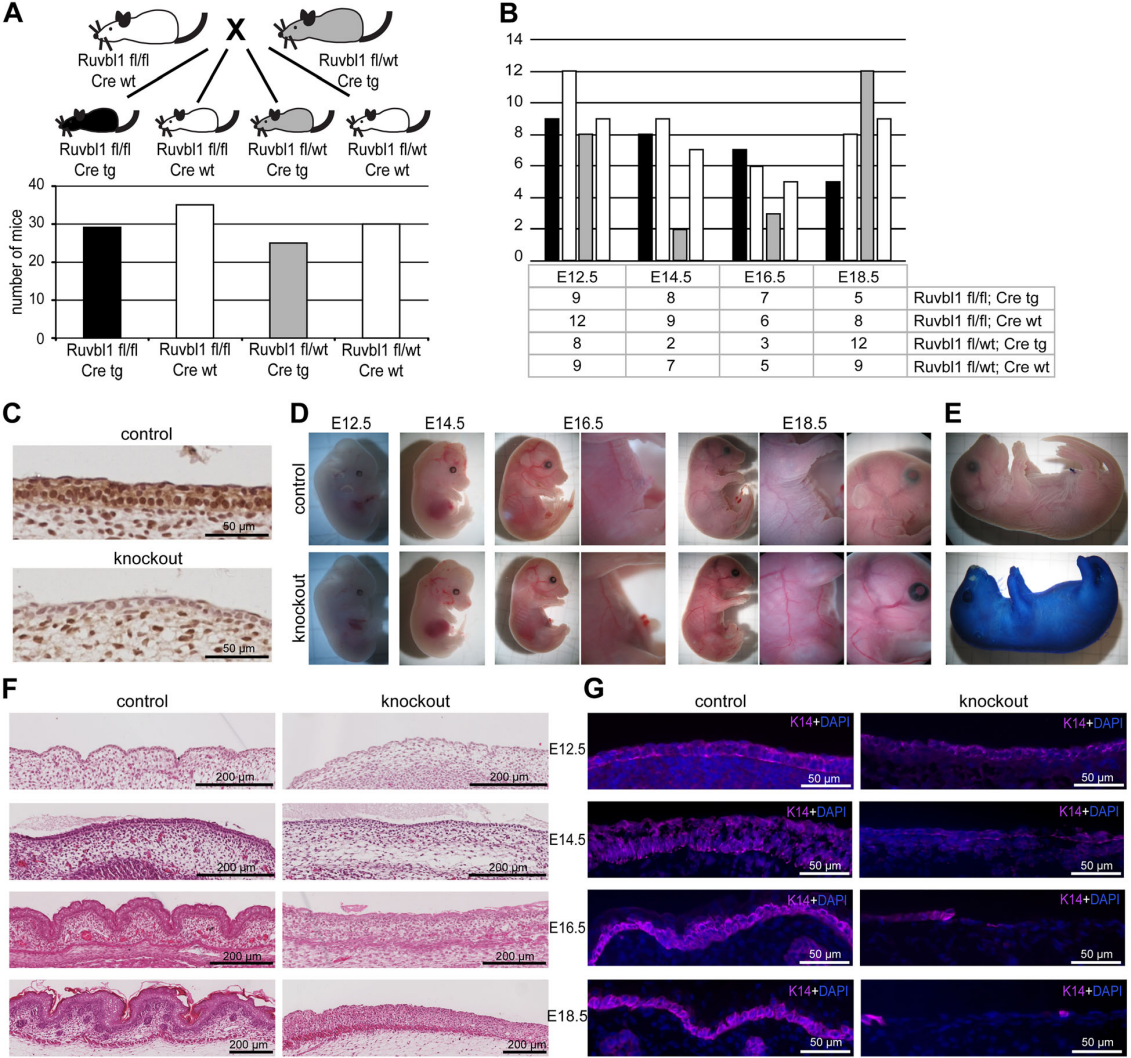
Epidermal knockout of *Ruvbl1* leads to severe skin development defects. **a** Although no living animals were observed, *Ruvbl1* epidermal knockout embryos (*Ruvbl1*^fl/fl^*K14:Cre*^tg^) are found in normal Mendelian ratios. The quantification summarizes mice from all embryonic stages examined. **b** Specific numbers of genotyped animals from different embryonic stages examined do not show a clear-cut pattern (E12.5, E14.5, E16.5, E18.5). **c** Immunostainings for Ruvbl1 on day E14.5 confirms loss of Ruvbl1 protein in *Ruvbl1* knockout mice. Control mice were *Ruvbl1*^fl/fl^*K14:Cre*^neg^. **d** Macroscopic images of *Ruvbl1*^fl/fl^*K14:Cre*^tg^ mice and control littermates demonstrate very thin skin and lacking skin folds in *Ruvbl1*^fl/fl^*K14:Cre*^tg^ mice during the course of development. **e** A skin barrier assay shows a strong defect in the skin barrier in *Ruvbl1*^fl/fl^*K14:Cre*^tg^ mice. **f** Histological HE staining show defects in general skin development in *Ruvbl1*^fl/fl^*K14:Cre*^tg^ mice starting in early embryonic development. **g** Immunostaining against K14 shows almost complete loss of epidermal tissue during late embryonic phases in *Ruvbl1*^fl/fl^*K14:Cre*^tg^ mice

To study the role of Ruvbl1 in the epidermis, we crossed the floxed *Ruvbl1* mouse line to the K14:Cre mouse line, resulting in a tissue-specific knockout of *Ruvbl1* in keratinocytes of the skin epidermis (Fig. 1a). Interestingly, we did not observe living *Ruvbl1*^fl/fl^*K14:Cre*^tg^ animals in our postnatal genotypings, although all other genotypes, including *Ruvbl1*^fl/wt^ *K14:Cre*^tg^ animals, developed normally and did not show obvious phenotypes during the follow-up of up to 2 years. *Ruvbl1* knockout animals developed in normal Mendelian ratios even until late embryonic stages (Fig. 1b) suggesting that epidermal *Ruvbl1* knockout results in perinatal mortality. Effective loss of the Ruvbl1 protein in the epidermis of *Ruvbl1* knockout animals was confirmed by immunohistochemistry stainings at day E14.5 (Fig. 1c). *Ruvbl1*^fl/wt^ *K14:Cre*^tg^ animals showed remaining epidermal expression of Ruvbl1 (Suppl. Fig. 1). Macroscopically, the *Ruvbl1*^fl/fl^*K14:Cre*^tg^ embryos at ages E12.5 and E14.5 did not show any obvious difference compared to the control littermates. From day E16.5 on, however, the skin of the *Ruvbl1*^fl/fl^*K14:Cre*^tg^ embryos seemed to be thinner and more stretched compared to controls. This was particularly striking when looking at the skin folds on the belly or the eyes of the embryos (Fig. 1d).

### Embryonic epidermal loss of *Ruvbl1* results in severe defects of epidermal development and function

To test functional epidermal properties of epidermal *Ruvbl1* knockout mice, we performed a skin barrier assay. In this assay, the uptake of toluidine blue indicates defects of the outside-in skin barrier. *Ruvbl1*^fl/fl^*K14:Cre*^tg^ clearly showed massive skin barrier defects at E18.5 (Fig. 1e). Histological analysis of *Ruvbl1*^fl/fl^*K14:Cre*^tg^ mice revealed defects in skin architecture and development already in very early stages (E12.5 and E14.5, Fig. 1f) with dramatic changes at later stages (E16.5 and E18.5). Skin appendages like hair follicles, which develop later, are lacking completely in *Ruvbl1* knockout mice. The stainings confirmed the stretched macroscopic appearance of the skin of the embryos. Immunofluorescence stainings of the skin using keratin-14 (K14) as marker for the epidermis showed a nearly complete loss of the K14-positive epidermal skin layer in *Ruvbl1* knockout embryos (Fig. 1f).

**Fig. 2:**
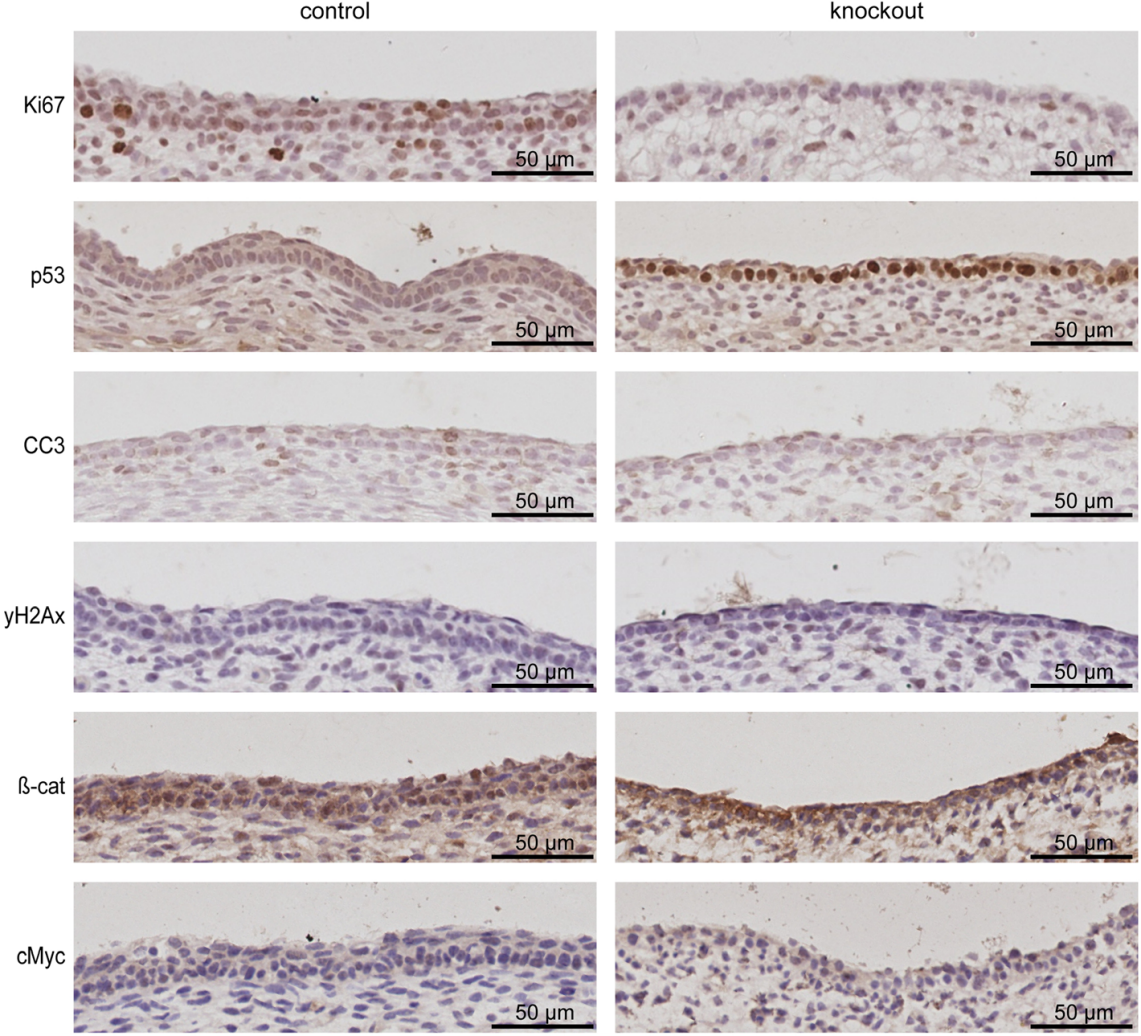
The pathophysiological mechanism of the Ruvbl1 epidermal phenotype may involve reduced proliferation and accumulation of p53. Immunohistochemical staining was performed on epidermal tissue of *Ruvbl1* knockout embryos and control littermates on day E14.5. Ki67 staining (upper panel), a marker for proliferation, was decreased in *Ruvbl1* epidermal knockout mice. In addition, an increased expression of p53 in the epidermal cells of *Ruvbl1* epidermal knockout mice was observed. There were no obvious differences in the staining of cleaved caspase 3, yH2AX, ß-catenin, or c-myc

Given the very early dramatic phenotype, we refrained from in-depth functional studies of signaling cascades in the epidermis in *Ruvbl1*^fl/fl^*K14:Cre*^tg^ mice. In first hypothesis-generating experiments, we evaluated markers of proliferation at an early stage of development. Indeed, *Ruvbl1*^fl/fl^*K14:Cre*^tg^ embryos on day E14.5 showed a reduced signal of Ki67-positive cells in the epidermis (Fig. 2). This was accompanied by a more pronounced signal of p53 staining suggesting an accumulation of the pro-apoptotic p53 with an activation of subsequent signaling in the epidermis of *Ruvbl1*^fl/fl^*K14:Cre*^tg^ embryos. Interestingly, however, we did not observe clearly altered expression of cleaved caspase 3 or of gammaH2AX, ß-catenin, or c-myc at day E14.5 (Fig. 2).

## Discussion

Mammalian epidermal development is a complex process. To generate the mature epidermis that consists of various layers to protect the body against dehydration or infection, signaling from multiple internal and external sources is integrated [1, 2]. In this manuscript, we show that the AAA+ superfamily protein Ruvbl1 is crucial for murine epidermal development. Targeted deletion of *Ruvbl1* during embryonic development of the epidermis results in severe defects of epidermal function and skin architecture showing that *Ruvbl1* is required for the normal development of murine epidermis and skin. *Ruvbl1*-deficient epidermal cells during development cannot survive and differentiate. Overall skin architecture, e.g., does not show normal development of hair follicles. The thin layer of cells during early embryonic epidermal development and the nearly complete loss of the K14-signal at later stages of development suggest that *Ruvbl1* is essential for survival of the epidermal basal cells from which all layers of epidermis are derived and that is required for the development of epidermal appendages.

Given the severity of this very early-onset phenotype, our data remain largely descriptive. Yet the findings can serve as a starting point for future more functional studies. Based on the phenotype and the first initial stainings, it is tempting to speculate that the observed phenotype is likely due to an imbalance of reduced proliferation and increased cell death potentially involving a dysregulation of p53 signaling early in the process. We did not observe evidence for increased detection of cleaved caspase-3 as a marker for apoptosis. However, it is well-known that p53 can regulate cell proliferation, cell survival, and cell death in response to different types of cellular stress in multiple ways [18]. Additional functional experiments on p53-associated signaling and, e.g., on the expression pattern of epidermal differentiation markers or of epigenetic regulators of epidermal development will be required but were beyond the scope of this manuscript with the focus of an initial report of the phenotype. The exact molecular mechanisms underlying the phenotype thus remain to be elucidated.

A link between Ruvbl1 and skin biology and disease has previously been established by the observation of Ruvbl1 and Ruvbl2 autoantibodies in a subcohort of patients suffering from systemic sclerosis but not from other connective tissue diseases [19, 20]. Patients in which the Ruvbl1/2 autoantibodies were detected showed more myositis and diffuse skin thickening and a high frequency of diffuse cutaneous involvement. These data may point to a role of Ruvbl proteins in the skin even after embryonic development. It may therefore also be interesting to study whether there is a role for *Ruvbl1* in epidermal and skin biology beyond the embryonic phase, thus whether the protein is also essential for the maintenance of epidermal structure and function. Again, more data will be required to shed light into the underlying biology. Given the early embryonically lethal phenotype of the knockout mouse and the severe phenotypes observed in different organs after targeted deletion, it seems unlikely that children with severe variants in *RUVBL1* would be born. Children with hypomorphic variants could potentially present with diverse syndromic phenotypes.

In summary, we identify *Ruvbl1* as a novel essential player in epidermal development. These observations add novel mammalian in vivo evidence to the growing body of literature on the important role of Ruvbl proteins in cellular proliferation and differentiation.

## Supplementary Information

**Additional file 1: Supplemental Figure 1.**. HE and Ruvbl1 immunohistochemistry staining of heterozygous mice: *Ruvbl1*^fl/wt^*K14:Cre*^tg^ mice show remaining expression of Ruvbl1 in immunohistochemistry staining.

## Data Availability

Original data is available upon reasonable request.
