# Supplementary material for: Targeted deletion of Ruvbl1 results in severe defects of epidermal development and perinatal mortality
Source: Mol Cell Pediatr. 2021 Feb 12;8:1. doi: 10.1186/s40348-021-00111-1 (PMC7881068; doi:10.1186/s40348-021-00111-1)
Supplement: Supplementary file 1 — Additional file 1: Supplemental Figure 1.. HE and Ruvbl1 immunohistochemistry staining of heterozygous mice: Ruvbl1fl/wtK14:Cretg mice show remaining expression of Ruvbl1 in immunohistochemistry staining. [file 40348_2021_111_MOESM1_ESM.docx]

**Supplementary Material:**


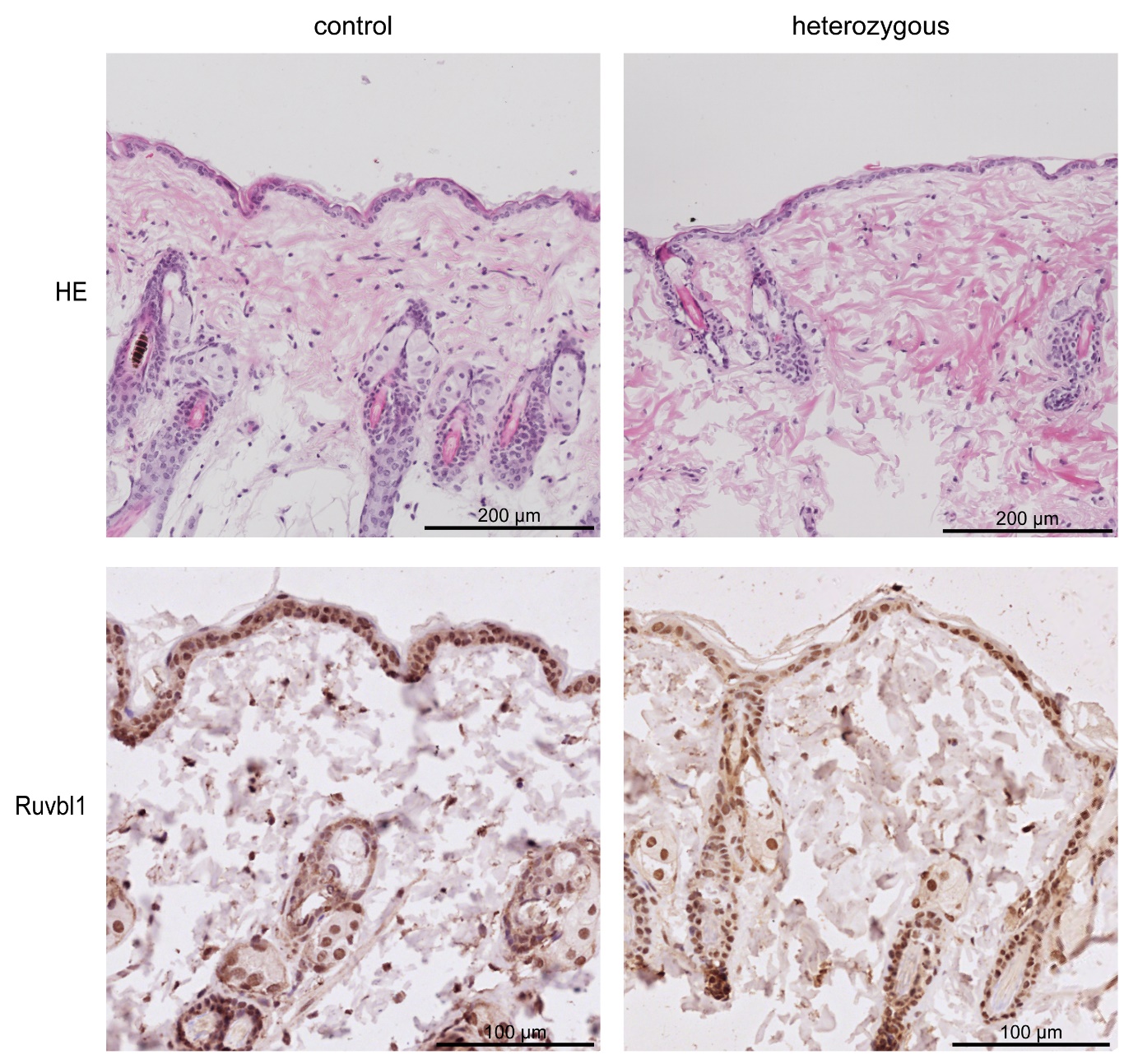


**Supplemental Figure 1:** HE and Ruvbl1 immunohistochemistry staining of heterozygous mice: *Ruvbl1*^fl/wt^*K14:Cre*^tg^ mice show remaining expression of Ruvbl1 in immunohistochemistry staining.
